# Combining OPM and lesion mapping data for epilepsy surgery planning: a simulation study

**DOI:** 10.1038/s41598-024-51857-3

**Published:** 2024-02-04

**Authors:** Stephanie Mellor, Ryan C. Timms, George C. O’Neill, Tim M. Tierney, Meaghan E. Spedden, The MELD Project Consortium, Hannah Spitzer, Mathilde Ripart, Kirstie Whitaker, Antonio Napolitano, Luca De Palma, Alessandro De Benedictis, Stephen Foldes, Kai Zhang, Wenhan Hu, Jiajie Mo, Marcus Likeman, Shirin Davies, Christopher Güttler, Matteo Lenge, Nathan T. Cohen, Yingying Tang, Shan Wang, Aswin Chari, Martin Tisdall, Nuria Bargallo, Estefanía Conde-Blanco, Jose Carlos Pariente, Saül Pascual-Diaz, Ignacio Delgado-Martínez, Carmen Pérez-Enríquez, Ilaria Lagorio, Eugenio Abela, Nandini Mullatti, Jonathan O’Muircheartaigh, Katy Vecchiato, Yawu Liu, Maria Eugenia Caligiuri, Ben Sinclair, Lucy Vivash, Anna Willard, Jothy Kandasamy, Ailsa McLellan, Drahoslav Sokol, Mira Semmelroch, Ane G. Kloster, Letícia Ribeiro, Clarissa Yasuda, Camilla Rossi-Espagnet, Khalid Hamandi, Anna Tietze, Carmen Barba, Renzo Guerrini, William Davis Gaillard, Xiaozhen You, Irene Wang, Sofía González-Ortiz, Mariasavina Severino, Pasquale Striano, Domenico Tortora, Reetta Kälviäinen, Antonio Gambardella, Angelo Labate, Patricia Desmond, Elaine Lui, Terence O’Brien, Jay Shetty, Graeme Jackson, John S. Duncan, Gavin P. Winston, Lars H. Pinborg, Fernando Cendes, J. Helen Cross, Torsten Baldeweg, Sophie Adler, Matthew J. Brookes, Konrad Wagstyl, Gareth R. Barnes

**Affiliations:** 1 Wellcome Centre for Human Neuroimaging, UCL Queen Square Institute of Neurology, University College London, London, WC1N 3AR UK; 2 Department of Neuroscience, Physiology and Pharmacology, University College London, London, UK; 3 Sir Peter Mansfield Imaging Centre, School of Physics and Astronomy, University of Nottingham, Nottingham, UK; 4 UCL Great Ormond Street Institute for Child Health, University College London, 30 Guilford St, London, WC1N 1EH UK; 5 Institute of Computational Biology, Helmholtz Center Munich, 85764 Munich, Germany; 6 The Alan Turing Institute, 96 Euston Rd, Somers Town, London, NW1 2DB UK; 7 Medical Physics Department, The Bambino Gesù Children’s Hospital, Rome, Italy; 8 Rare and Complex Epilepsies, Department of Neurosciences, Bambino Gesù Children’s Hospital, IRCCS, Rome, Italy; 9 Department of Neurosciences, Neurosurgery Unit, Bambino Gesù Children’s Hospital, IRCCS, Rome, Italy; 10 Barrow Neurological Institute at Phoenix Children’s Hospital, 1919 E Thomas Rd, Phoenix, AZ 85016 USA; 11 Department of Neurosurgery, Beijing Tiantan Hospital, Capital Medical University, Beijing, China; 12 Bristol Royal Hospital for Children, Upper Maudlin St, Bristol, UK; 13 School of Psychology, Cardiff University Brain Research Imaging Centre, Cardiff, UK; 14 The Welsh Epilepsy Unit, Cardiff and Vale University Health Board, University Hospital of Wales, Cardiff, UK; 15 Charité University Hospital, Berlin, Germany; 16 Neuroscience Department, Children’s Hospital Meyer-University of Florence, Viale Pieraccini 24, 50139 Florence, Italy; 17 Center for Neuroscience Research, Children’s National Hospital, The George Washington University School of Medicine, Washington, DC USA; 18 Department of Neurology, West China Hospital of Sichuan University, Chengdu, China; 19 Epilepsy Center, Cleveland Clinic, Cleveland, OH USA; 20 Department of Neurology, School of Medicine, Epilepsy Center, Second Affiliated Hospital, Zhejiang University, Hangzhou, China; 21 Great Ormond Street Hospital, NHS Foundation Trust, London, UK; 22 Department of Neuroradiology, Core Facility, Hospital Clinic Barcelona and Magnetic Resonance Imaging, IDIBAPS, Barcelona, Spain; 23 Centro de Investigación Biomédica en Red de Salud Mental, CIBERSAM, Barcelona, Spain; 24 Magnetic Resonance Imaging, Core Facility, IDIBAPS, C/Rossello 149, 08036 Barcelona, Spain; 25 Department of Neurosurgery, Hospital del Mar, Barcelona, Spain; 26 Department of Neurology, Hospital del Mar, Barcelona, Spain; 27 IRCCS Istituto Giannina Gaslini, Via Gaslini 5, 16147 Genova, Italy; 28 Center for Neuropsychiatry and Intellectual Disability, Psychiatrische Dienste Aargau AG, Königsfelderstrasse 1, 5120 Windisch, Switzerland; 29 Institute of Psychiatry, Psychology and Neuroscience, 16 De Crespigny Park, Camberwell, London, SE5 8AB UK; 30 Department of Forensic and Neurodevelopmental Sciences, Institute of Psychiatry, Psychology and Neuroscience, MRC Centre for Neurodevelopmental Disorders, King’s College London, London, UK; 31 Department of Perinatal Imaging and Health, St. Thomas’ Hospital, King’s College London, London, UK; 32 Department of Neurology, University of Eastern Finland, Kuopio, Finland; 33 Department of Medical and Surgical Sciences, Neuroscience Research Center, Magna Graecia University of Catanzaro, Viale Europa, 88100 Catanzaro, Italy; 34 Department of Neuroscience, Central Clinical School, Level 6 The Alfred Centre, Monash University, 99 Commercial Road, Melbourne, VIC 3004 Australia; 35 Central Clinical School, Alfred Centre, 99 Commercial Rd, Melbourne VIC 3004, Australia; 36 Department of Neurology, Alfred Health, Commercial Road, Melbourne, Australia; 37 Royal Hospital for Children and Young People, Little France Crescent, Edinburgh, EH16 4TJ UK; 38 The Florey Institute of Neuroscience and Mental Health, 245 Burgundy St, Austin Campus, Heidelberg, Victoria 3071 Australia; 39 Neurobiology Research Unit, Copenhagen University Hospital - Rigshopsitalet, Copenhagen, Denmark; 40 Department of Neurology, University of Campinas - UNICAMP, Rua Vital Brasil, 251, Cidade Universitária, Campinas, SP 13083-888 Brazil; 41 Brazilian Institute of Neuroscience and Neurotechnology (BRAINN), Campinas, SP Brazil; 42 Neuroradiology Unit, IRCCS Bambino Gesù Children’s Hospital, Rome, Italy; 43 Department of Neurosciences, Rehabilitation, Ophthalmology, Genetics, Maternal and Child Health, University of Genova, Genova, Italy; 44 Kuopio Epilepsy Center, Neurocenter, Kuopio University Hospital, Kuopio, Finland; 45 Institute of Neurology, Department of Medical and Surgical Sciences, Magna Graecia University, Viale Europa, 88100 Catanzaro, Italy; 46 Department of BIOMORF, Neurology Unit, University of Messina, Messina, Italy; 47 Department of Radiology, Royal Melbourne Hospital, University of Melbourne, Parkville, VIC 3050 Australia; 48 Monash University, 99 Commercial Road, Melbourne, VIC 3004 Australia; 49 Department of Medicine, The Royal Melbourne Hospital, Royal Parade, Parkville, VIC 3052 Australia; 50 Department of Neurology, Austin Health, Heidelberg, Australia; 51 UCL Queen Square Institute of Neurology, Queen Square, London, WC1N 3BG UK; 52 Department of Medicine, Division of Neurology, Queen’s University, Kingston, Canada; 53 Department of Neurology, Epilepsy Clinic, Copenhagen University Hospital - Rigshopsitalet, Copenhagen, Denmark; 54 Young Epilepsy, Lingfield, UK

**Keywords:** Magnetoencephalography, Epilepsy, Biomedical engineering

## Abstract

When planning for epilepsy surgery, multiple potential sites for resection may be identified through anatomical imaging. Magnetoencephalography (MEG) using optically pumped sensors (OP-MEG) is a non-invasive functional neuroimaging technique which could be used to help identify the epileptogenic zone from these candidate regions. Here we test the utility of a-priori information from anatomical imaging for differentiating potential lesion sites with OP-MEG. We investigate a number of scenarios: whether to use rigid or flexible sensor arrays, with or without a-priori source information and with or without source modelling errors. We simulated OP-MEG recordings for 1309 potential lesion sites identified from anatomical images in the Multi-centre Epilepsy Lesion Detection (MELD) project. To localise the simulated data, we used three source inversion schemes: unconstrained, prior source locations at centre of the candidate sites, and prior source locations within a volume around the lesion location. We found that prior knowledge of the candidate lesion zones made the inversion robust to errors in sensor gain, orientation and even location. When the reconstruction was too highly restricted and the source assumptions were inaccurate, the utility of this a-priori information was undermined. Overall, we found that constraining the reconstruction to the region including and around the participant’s potential lesion sites provided the best compromise of robustness against modelling or measurement error.

## Introduction

Approximately 0.6% of the population are living with epilepsy^1^. Around a third of patients do not respond to medication^2^. These patients may be considered for surgery, with the aim of removing the region of the brain causing the seizures^3–6^. Determining this area is a key part of presurgical planning. Multiple neuroimaging techniques are often used, including Magnetic Resonance Imaging (MRI), single-photon emission computed tomography (SPECT) and scalp electroencephalography (EEG)^7^. It is relatively common for more than one candidate site to be identified. Intracranial EEG (iEEG) may be undertaken, with electrodes implanted in these candidate regions, to confirm the expected epileptogenic focus or choose between potential candidates^8,9^. This is an effective but invasive method to determine the region for surgical resection^10^.

Magnetoencephalography (MEG) has been suggested as a potential method to guide or avoid intracranial EEG^11–15^. MEG is a neuroimaging technique where the magnetic field induced by neuronal currents is measured^16,17^. The origin of the MEG and EEG signals are the same, but unlike the electrical component measured in EEG, the magnetic component is minimally distorted by the skull. This means that high spatial resolution can be obtained non-invasively with MEG. There is strong evidence that removal of the epileptogenic focus as found in MEG is correlated with good surgical outcomes^18,19^. However, MEG systems have traditionally been optimised for compliant adults and so are sub-optimal for the younger population who would benefit the most from surgery^20^.

Optically Pumped Magnetometer (OPM) based MEG (OP-MEG) uses new magnetic sensors to overcome many of the previous limitations of MEG. These sensors do not require cryogenic cooling and so can be worn directly on the scalp^21–23^. This increases the signal at the sensors and, perhaps more importantly for a clinical setting, reduces cost and allows participants to move while wearing the system. These latter points potentially increase patient compliance^24–26^ and accessibility of the technology.

There are, however, some additional challenges with OP-MEG. The flexibility of sensor placement means that, depending on the helmet used to hold the sensors on the head, there may be uncertainty in the sensor positions and they may move relative to one another^27^. Additionally, many OPMs operate in the spin exchange relaxation free (SERF) regime and so operate best at magnetic fields close to zero (approximately $\pm$ 2 nT)^28–32^. Changes in the background magnetic field from, for example, movement or urban noise, can introduce errors in the OPM gain, phase and orientation of the sensitive axis^33,34^. Here we consider how OP-MEG might be used for presurgical planning in epilepsy in simulation, while considering the impact of these different error sources. We focus on whether OP-MEG can identify the epileptogenic focus from potential sites identified with other clinical assessments, effectively fulfilling the role often taken by iEEG.

As OP-MEG is a new technology within the context of epilepsy, OP-MEG recordings exist for only a small number of patients^35–39^. We have therefore opted for a simulation approach. This has allowed us to consider epileptogenic lesions from over 500 patients. To ensure realistic lesion sites and sizes, we simulated activity from lesions identified in the Multi-centre Epilepsy Lesion Detection (MELD) Project^40^. The aim of the MELD FCD project is to use Machine Learning methods to improve detection of Focal Cortical Dysplasias (FCDs) from MRI. FCDs are focal areas of abnormal neuronal organisation which may lead to epilepsy and are a leading cause of drug-resistant epilepsy in the paediatric population^41–43^. Previous results from the MELD study have shown that the spatial distribution of these lesions is not uniform, but that a greater number are located in the brain’s frontal and temporal lobes than elsewhere^44^. Often, multiple potential sites are identified for each patient. Here we use the information from the MELD study to simulate OP-MEG recordings from potential lesions in a large number (> 1000) of anatomically feasible locations and ask whether we can differentiate between potential sites within a patient.

What is not known, but is an active area of research, is which part (if any) of an FCD causes seizures^45^. The abnormal electrical activity may originate predominantly from the centre of the anatomically identified lesion mass or may be from the surrounding tissue^46–48^. This is likely different for different patients. Consequently, as well as sensor errors, we also consider the impact of errors in our assumptions about the source model on the ability of OP-MEG to differentiate the correct lesion.

In this paper, we will show that the use of anatomical priors can improve OP-MEG source estimation in this context of epilepsy surgery. We will examine how errors in assumed source location, and sensor position, orientation and gain degrade these source estimates. Lastly, we will investigate the impact of the distance between the lesions on source estimation performance. While we focus the discussion around epilepsy, many of the results will be applicable to the wider use of OP-MEG where there is a strong prior hypothesis for the involved brain regions.

## Methods

### OP-MEG data simulation

**Figure 1:**
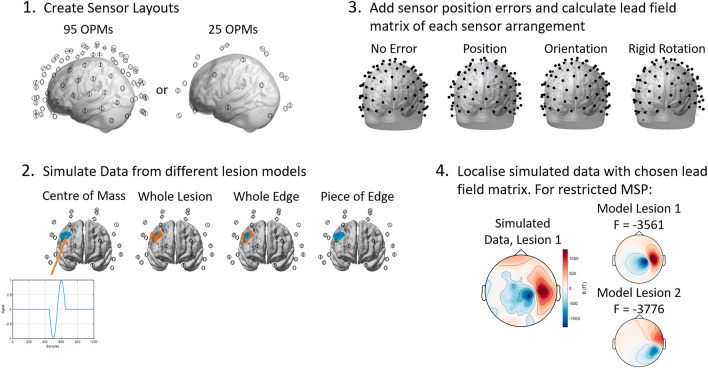
Data simulation and model comparison pipeline. Sensor layouts were created with 95 and 25 OPMs. The lead fields for the Nolte single shell model were calculated with the sensors in their original positions and then with the sensor positions or orientations changed. OP-MEG data at the sensors (in their original positions) were simulated with 4 different source models for each lesion: the centre of mass (COM) of the lesion, the whole lesion active, the whole edge or boundary of the lesion and a piece of the lesion edge. The blue area in panel 2 shows the lesion map for an example lesion, the orange dots are the mesh vertices simulated as being active in each condition. The simulated data was then localised using three different methods: an empirical Bayesian Beamformer (EBB), uninformed of the potential lesion sites, a loosely constrained implementation of MSP, with a patient’s anatomical lesions and the regions around them as priors, and a highly restricted version of MSP with the centre of mass of a patient’s anatomical lesions as priors. For both MSP inversions, each of a patient’s lesions was modelled separately and the Free Energy of the models compared. The lesion/prior with the higher Free Energy was retained.

We simulated OP-MEG data from two different sensor arrangements in SPM12^49^. The two sensor arrangements differed only in the separation between sensors: in one, it was 32 mm resulting in 95 OPMs (or 190 channels); while in the other it was set at 64 mm, equating to 25 OPMs (50 channels). In each case, the sensors were uniformly distributed and the OPM channels were offset from the scalp by 8.7 mm (see Fig. 1). 8.7 mm was chosen as it is the expected distance between the centre of the OPM cell and the scalp for our OPM system, incorporating the distance between the edge of the OPM and the centre of the cell (6.2 mm), an offset to insulate the scalp from the heat of the sensor (1.5 mm) and an offset to account for the curvature of the head (1 mm). Dual-axis sensors were simulated, meaning that for each simulated OPM sensor two channels were simulated, with one channel radial to the scalp while one was arbitrarily oriented tangentially to it. Dual-axis sensors were chosen (rather than increasingly common triaxial sensors) because all current, commercially available OP-MEG systems use sensors with at least two axes. Further details on how the sensor layouts were generated are given in Ref.^50^ and the code can be found here: (https://github.com/spm/spm/blob/main/toolbox/MEEGtools/spm_opm_sim.m).

The neural source of the simulated activity was chosen based on the lesion sites predicted as part of the Multi-Centre Epilepsy Lesion Detection (MELD) project^40^. These predictions were generated by an artificial neural network, trained on the MELD multi-centre dataset to identify FCDs from features of the cortical surface including cortical thickness, grey-white contrast, mean curvature, sulcal depth and intrinsic curvature. The network was trained on FCD lesions delineated on a T1-weighted MRI by a neuroradiologist, neurologist or experienced epilepsy researcher. Further details on how the potential lesion sites were identified with MELD can be found here^40^.

**Figure 2:**
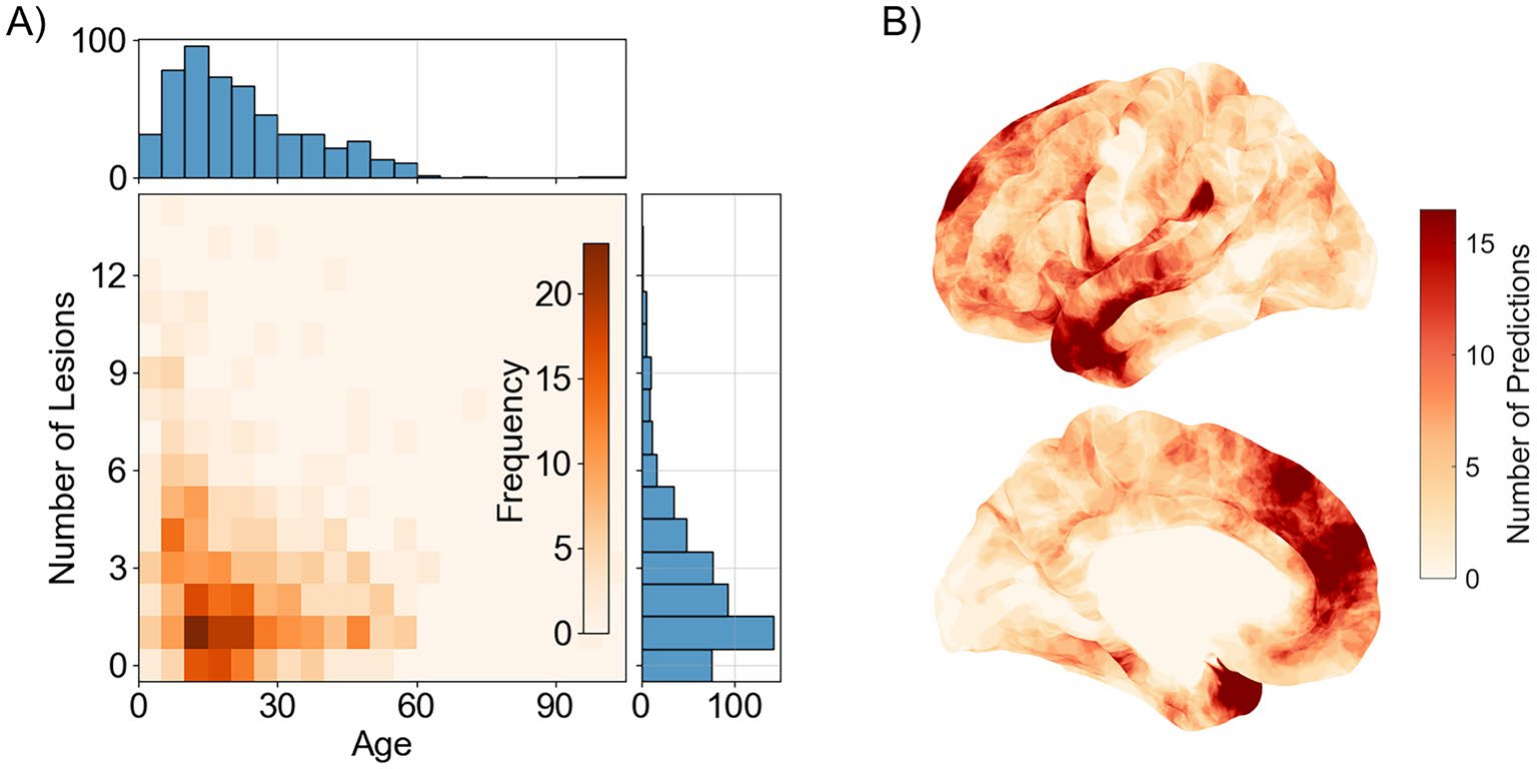
(**A**) Joint histogram of age and number of predicted lesions for participants in the MELD dataset. While the modal average number of predicted lesions is one, the median number is two. (**B**) Distribution of lesion predictions across the cortical surface. There is a higher density of lesions in the temporal or frontal lobes than elsewhere. Based on Ref.^44^.

At the time of generating this OP-MEG dataset, this dataset included 538 patients with a median of 2 potential lesion locations per patient. The distribution of the number of lesions per patient and patient age in the dataset is shown in Fig. 2A. Figure 2B shows the spatial distribution of simulated lesions. These lie predominantly in the temporal and frontal lobes. For every patient where 2 or more potential lesion sites were identified by the MELD algorithm, we simulated activity at each predicted lesion site separately, assuming a situation where one lesion was true and is causing epileptogenic activity while the others are inert. I.e. if a patient had 2 lesions, we created 4 datasets, one for each lesion and sensor arrangement. This resulted in simulating OP-MEG data for 1309 possible lesions.

The lesion maps from the MELD study were all expressed in the coordinate frame of the FreeSurfer average mesh. Given the large number of lesion locations (1309), for computing efficiency, before simulating OP-MEG data in SPM, the FreeSurfer average mesh was downsampled by a factor of 100 and then used as the source space in SPM. The number of mesh vertices decreased from 327,684 to 3280, with an average vertex separation going from 0.547 $\pm$ 0.001 mm to 3.878 $\pm$ 0.024 mm.

At each lesion site, we simulated electrical activity on the cortical mesh in 4 possible scenarios: (i) a single dipolar source at the centre of mass (COM) of the lesion; (ii) multiple, identical dipolar sources at every vertex within the whole lesion (WL); (iii) multiple, identical dipolar sources around the entire boundary of the lesion (whole edge (WE)); and (iv) a single dipolar source on the boundary of the lesion (piece of edge (POE)). Further details on how this was done are given in the Supplementary Material and the simulation code can be found here (https://github.com/stephaniemellor/opm_meld_simulations). In all cases, the sensor SNR (simulating Gaussian brain noise) was set at − 20 dB, the default value in SPM. After the temporal projection, which is performed by default in SPM in all of the source reconstruction methods tested here, the effective SNR of the signal was − 0.1 $\pm$ 0.1 dB. The fixed SNR avoided confounds due to amplitude changes when considering large vs. small patches of active cortex, although it does artificially inflate the expected SNR of sources deep in the brain by comparison with shallow sources (see discussion). The forward model determining how neural currents are observed at the sensors was the Nolte single shell model^51^. Dipole orientations were modelled normal to the cortical mesh. The form of the simulated activity was a single wavelength of a cosine wave with a wavelength of 200 ms at the centre of a 1 s long trial (as shown in Fig. 1).

### Simulated sensor errors

We simulated sensor position and orientation errors by updating the original sensor layouts and then recalculating the forward model. We then used this distorted forward model to localise the previously simulated data (simulated with the correct forward model). This meant that all lesions had the same position and orientation errors and minimised the number of calculations required. By contrast, gain errors were applied by multiplying the data from each channel by a number randomly selected from a zero-mean Gaussian distribution with a given variance, and so were different for each lesion.

Two types of sensor error were introduced: “flexible” type errors where sensors moved independently and “rigid” rotation errors where the entire sensor array rotated as a rigid body. To add flexible errors, for each sensor, we selected a position or orientation error from a zero-mean Gaussian distribution with a given standard deviation. The standard deviation was varied between 0 and 10 mm in steps of 2 mm (with an extra measurement at 5 mm) for position errors, and between 0 and 20 degrees in steps of 5 degrees for orientation errors. Sensor gain errors were also added, where each channel (bearing in mind that there are two channels per sensor) was treated independently. Here the simulated data were multiplied by a factor selected from a zero-mean Gaussian distribution with a standard deviation varying from 0 to 5% of the signal maximum in steps of 1%. To add rigid errors, we rotated the helmet around a randomly selected axis by a given degree. The axis was changed for each rotation. The rotation angle was varied between 0 and 20 degrees in steps of 5 degrees. This rigid rotation is more akin to the co-registration errors observed in cryogenic MEG where the head moves within a fixed helmet.

### Source inversion

To estimate the origin of the simulated activity, in all cases, the Nolte single shell forward model was used and the downsampled FreeSurfer average cortex was the source space. Three source inversion methods were used. First, an Empirical Bayesian Beamformer (EBB) was run in SPM across the entire source space^52^. Secondly, we ran a loosely constrained form of multiple sparse priors (MSP)^53^ iteratively for each of a patient’s lesions, each time restricting the potential source space to only the 100 cortical mesh vertices nearest to the centre of mass (COM) of a particular lesion (mean volume 23.2 cm^3^, 5th percentile: 17.3 cm^3^, 95th percentile: 31.6 cm^3^) (details are given in section "Loose priors"). Thirdly, a more restricted form of MSP was run, with the only priors being the single dipoles corresponding to the centre of mass (COM) of each of the patients’ lesions (see section "Restricted priors" for details).

To evaluate these priors or models we used the negative variational Free Energy metrics (henceforth referred to as Free Energy) provided in SPM. Free Energy is an approximation of model evidence, typically expressed in log units, and can be defined as the model accuracy minus its complexity^54^, meaning that it rewards accuracy in describing the observed data while penalising overly complex models which may be overfitting. A higher Free Energy value therefore indicates a better fit of the data. A (log) Free Energy difference between models of 3 implies that one model is 20 (${\approx e}^{3}$) times more likely than the other.

To compare the reconstruction methods, we looked at the percentage of lesions which could be correctly differentiated from all of a patient’s other potential lesion sites. How we determined whether the correct lesion was found was slightly different for each method and is described below. Due to the distribution of potential lesion sites per patient (see Fig. 2A), at chance level, 24.4% (95% confidence interval: 24.3–24.5%) of lesions would be correctly differentiated.

#### Uninformed reconstruction

We used EBB to test performance without prior information. The full 1 s of data was used in the reconstruction, which was bandpass filtered between 0 to 256 Hz using the discrete cosine transform in SPM. A Hanning window was used to minimise edge effects. To evaluate the performance, we looked at the location of the peak of the estimated neural current density. We recorded the distance between this peak and the mean position of the simulated cortical mesh vertices. If this peak was closer to the simulated lesion than any other possible lesion location for the same patient, the lesion was considered to be correctly identified.

#### Anatomically informed MSP

In addition to EBB, we ran two restricted versions of MSP. MSP is based on the Bayesian framework, where the posterior model evidence for the MEG source distribution is optimised based on a set of source priors. Here, we restricted those priors based on the anatomical lesion predictions. As in the EBB reconstruction, the entire 1 s trial window was used in the reconstruction and the data were bandpass filtered between 0 and 256 Hz. We removed the spatial decomposition usually used within SPM (which reduces the effective channel count through a principal component analysis), in order to fairly compare cases where the sensors had been moved.

##### Loose priors

For a loosely constrained version of MSP, we limited the potential solutions to within the nearest 100 mesh vertices of the COM of each lesion (mean volume 23.2 $\pm$ 0.2 cm^3^). That is, we used 100 neighbouring dipole sources as priors to test each potential lesion location. MSP iteratively optimizes model evidence (Free energy) to return a weighted sum of these priors to explain the OP-MEG data. The final Free energy value can be used to judge the quality of the fit. From all the potential reconstructions (e.g. if the patient had 12 potential lesion sites, there were 12 reconstructions to choose between), the solution with the highest Free Energy value was used. The peak estimated current in this accepted solution was then found and, as with the EBB reconstruction, if this peak current location was closer to the mean location of the true, simulated mesh vertices than the mean location of the vertices describing any of the other potential lesion sites in that patient, the lesion was marked as correctly identified.

##### Restricted priors

In the classical implementation of MSP^53^, a weighted sum of the priors is optimised to describe the MEG data. Here, rather than testing many priors, we used the MSP framework to test a single prior at a time. We created one source prior for a single dipole at the COM of each of a patient’s lesions and used the Free Energy of the resulting reconstructions to test whether there was stronger evidence for the prior at the location of the simulated lesion compared to the patient’s other potential lesion sites.

## Results

We first consider the impact of different source and sensor errors on the Free Energy differences between priors for the restricted MSP reconstruction. We describe how we assessed the likelihood of different lesions being active based on this prior knowledge of their potential locations. We then look at how an algorithm (EBB) without such a-priori information would perform, as well as the MSP solution based on loose priors. The results here all consider the minimal sensor spacing (and hence the maximum number of sensors) tested; results for the double spacing (approximately quarter the sensor number) can be found in the Supplementary Material.

**Figure 3:**
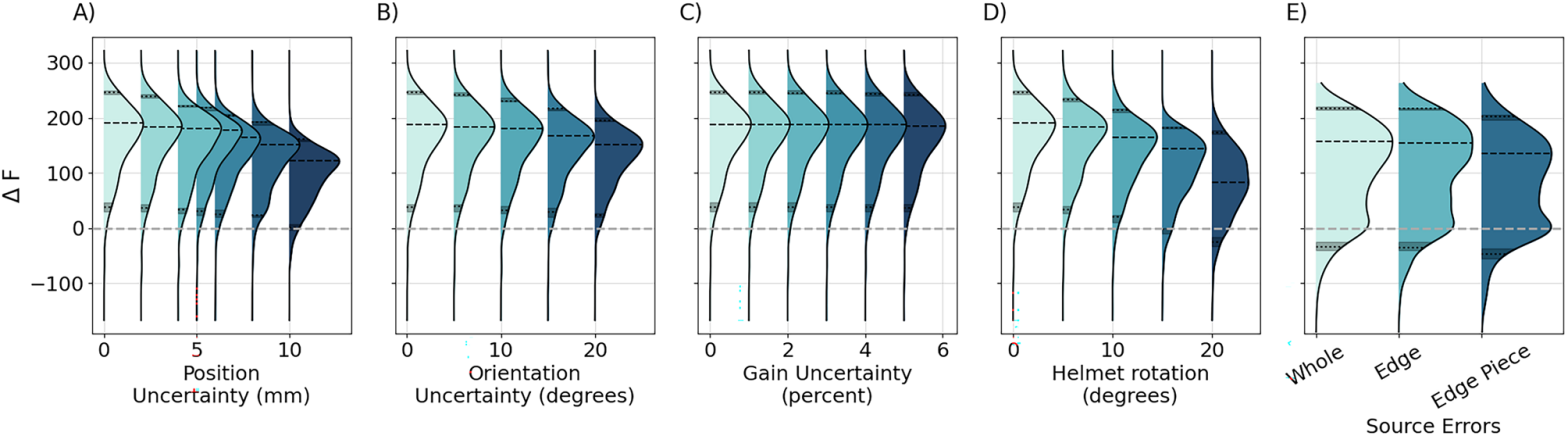
Distribution of the Free Energy difference between the correct (simulated) lesion model and the alternative lesion model with the next highest Free Energy ($\mathrm{\Delta F}$) for all 1309 lesions from the restricted reconstruction. Values of $\mathrm{\Delta F}$ below 0 indicate that an incorrect lesion would be selected. The maximum of the distribution and 5th and 95th percentile values are marked with dashed black lines. In (**A–D**), different sensor errors are shown. Flexible type errors (position (**A**), orientation (**B**) and gain uncertainties (**C**)) have a small effect by comparison with rigid helment rotation (**D**). Source errors (**E**), show the largest negative skew in the distribution of $\mathrm{\Delta F}$. In other words, for the scenarios tested here, incorrect source models undermine the correct lesion choice to a much greater degree than incorrect sensor models.

Figure 3 shows the distribution of Free Energy differences ($\Delta F$) from the potential lesion locations when only the COM of each of the patient’s lesions was compared (the highly restricted MSP reconstruction). If $\Delta F>0$, the correct lesion would be selected as active. In panels A-B, errors in knowledge of the sensor location and orientation which may occur in a flexible array are considered. It is striking that, even for relatively large errors, the correct lesion choice (or $\Delta F>0$) is robust. This is possibly due to both the independence of these errors (across sensors) and the prior information provided (i.e. it is a choice between fewer than 14 potential sites). Similar behaviour is observed for the addition of random gain errors (Fig. 3C).

Figure 3D shows the effect of co-registration errors in the case of a rigid helmet with unmodelled rotations. The lesion choice makes the algorithm relatively robust to small co-registration errors ($\le 10$ mm). However, in contrast to the flexible helmet case, performance was more sensitive to the size of the rigid rotation.

Figure 3E shows the influence of inaccurate assumptions about the distribution of current across the lesion. In this case, there is a notable drop in performance. This implies that, even for perfect forward models, if the source reconstruction is too restrictive and does not represent the source level activity, this will undermine the correct lesion choice.

We now compare this restricted prior comparison with loosely constraining the solution around the expected anatomical region of interest and with simply source localising with no prior information. In these scenarios, we judge the outcome to be successful if the estimated current density peak is closer (in Euclidean distance) to the correct simulated lesion site than the others. We found that in all conditions, including anatomical information in the reconstruction benefitted the outcome.

**Figure 4:**
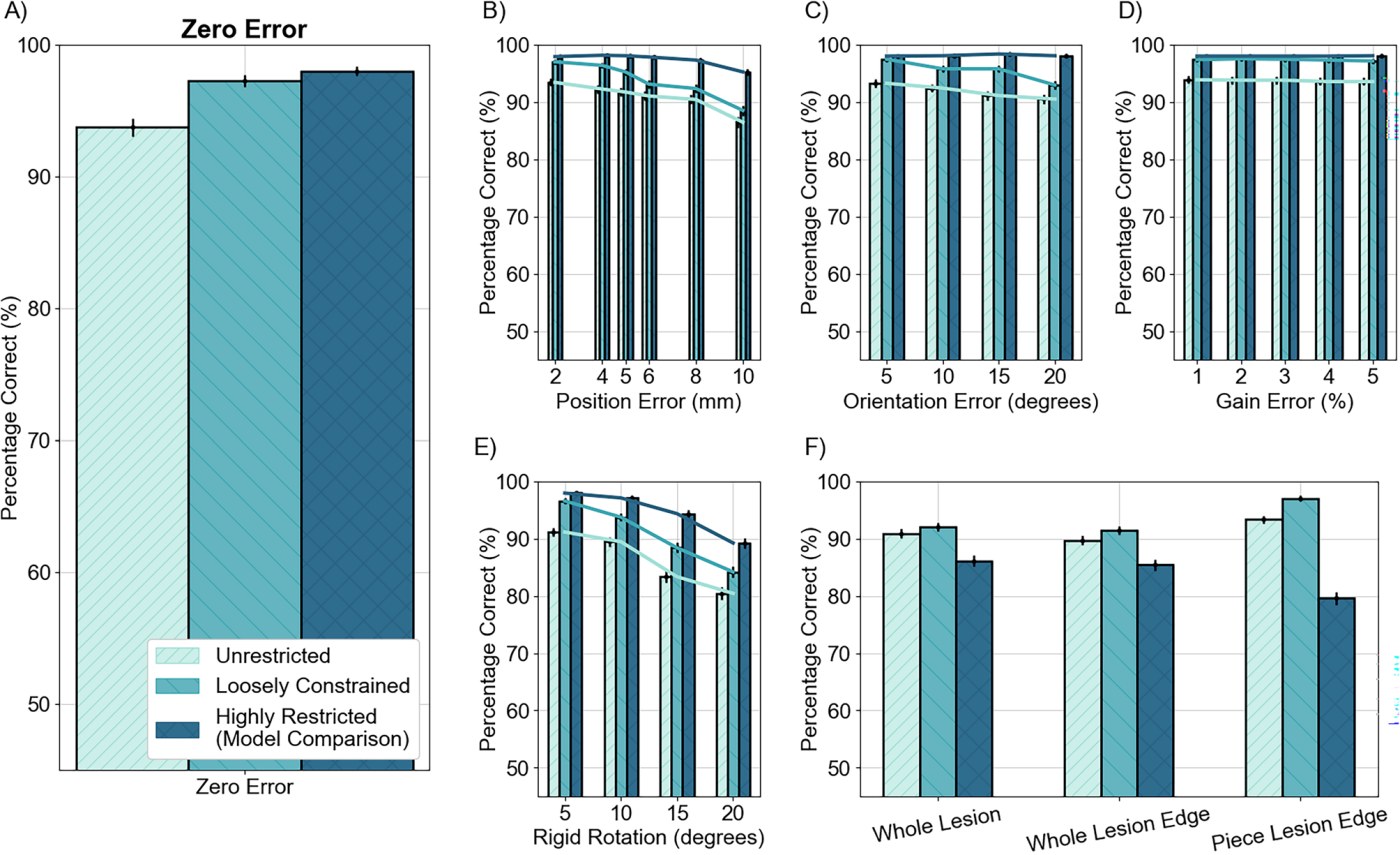
Comparison of the reconstruction methods tested: unrestricted EBB, MSP loosely constrained around the lesion site and MSP restricted to the centre of mass of the lesion. The percentage of lesions which were correctly differentiated from any of the patient’s other lesions is shown against the different errors added. The highly restricted method performs best when the source model is the same as the simulated source (i.e. the COM of the lesion was used both to simulate and model the data). When the model was not representative of the simulated sources, the loosely constrained method performed the best. In all cases, all methods performed considerably higher than the chance level of 24.4%.

When the source model matches the simulated sources, the highly restricted model comparison outperforms the uninformed and loosely restricted inversions (Fig. 4A–E). In the flexible array (independent sensor errors, Fig. 4B and C), the percentage of lesions correctly identified with all methods falls as random position and orientation errors are increased, but the highly restricted, model comparison method is comparatively robust to these errors. The performance of all methods decreased more rapidly with rotation errors of a rigid sensor array (Fig. 4E). Independent gain errors (Fig. 4D) had the least effect on the correct lesion choice.

When the simulated current distribution and expected source model does not agree however, the performance of the restricted model comparison method decreases considerably (Fig. 4F). The decrease in percentage of lesions correctly identified by the highly restricted reconstruction is largest when only a piece of the edge was simulated. It seems that this is the case as none of the current density priors we are testing between reflects the true underlying source distribution (e.g. the simulated data is based on the lesion edge, but the prior only tests the lesion centre of mass). As a result, the uninformed and loosely constrained reconstructions outperform the highly restricted model comparison method in this scenario, as they are able to accommodate this inaccuracy. In all cases, the loosely constrained reconstruction outperformed the unrestricted, uninformed reconstruction.

**Figure 5:**
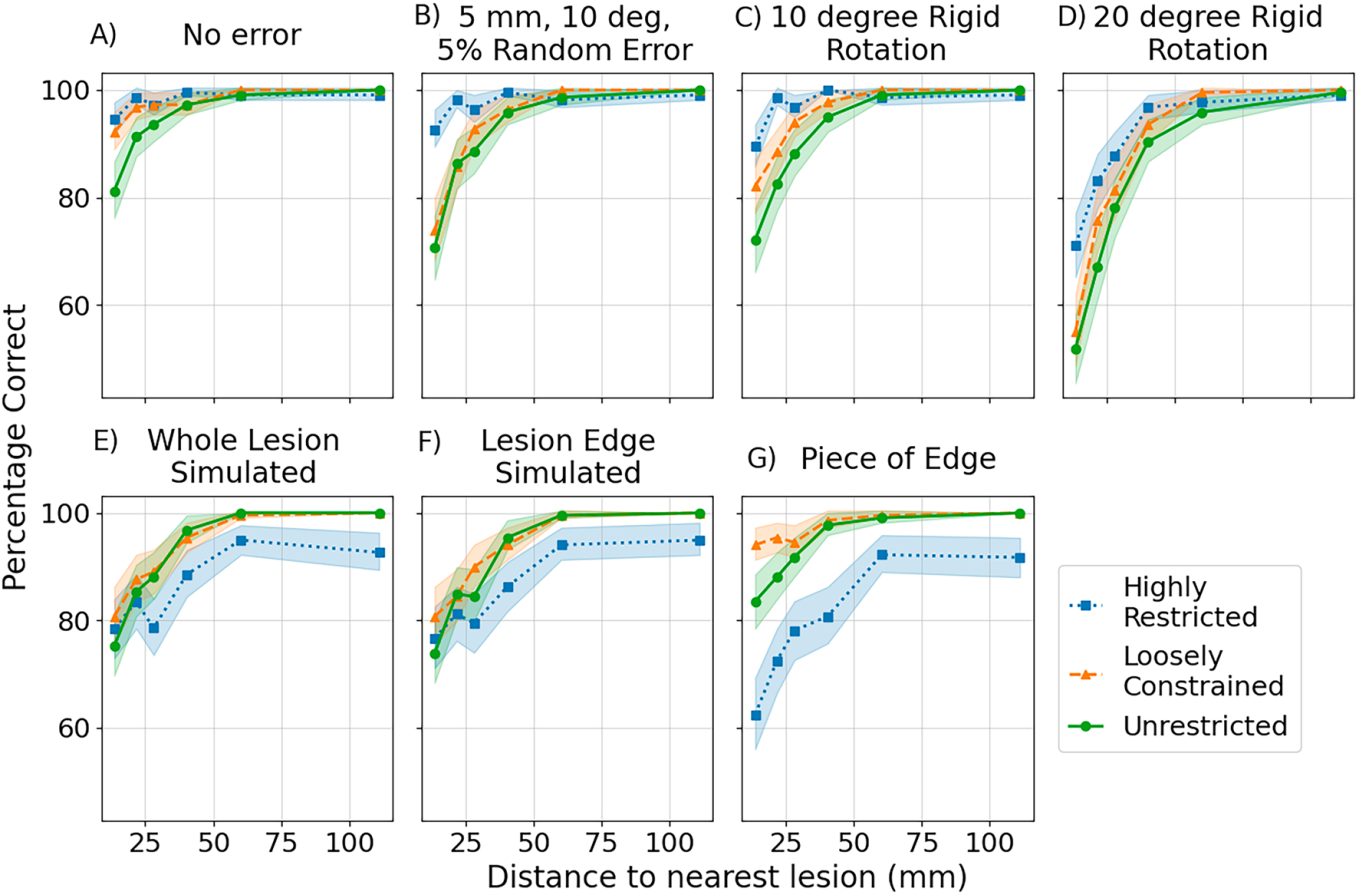
Relationship between the percentage of lesions correctly identified and distance to the nearest lesion from the same patient (i.e. the nearest alternative potential lesion site). All three reconstruction methods are shown for a selection of sensor and source errors. Each datapoint represents the percentage of lesions correctly identified within a distance range (or bin). The distance ranges are sampled so that there are equal numbers of lesions in each distance range (or bin). The points indicate the centre of each bin. The shaded area gives the 95% confidence interval of the percentage correct in each bin, estimated by bootstrapping with the scipy stats toolbox. (**A**) No errors or uncertainties have been added. (**B**) Random errors of 5 mm to the OPM positions, 10 degrees to the OPM orientations and 5% to the OPM channel gains have been added. (**C**,**D**) 10 degree (**C**) and 20 degree (**D**) rigid helmet rotations. (**E**–**G**) Sensor errors were set to zero and source errors were introduced, with the whole lesion (**E**), edge of the lesion (**F**) and a single vertex on the edge of the lesion (**G**) simulated while the COM of the lesion was used to model the lesion in the highly restricted reconstruction.

To consider the factors that may make a lesion easier to separate from other potential lesion sites, Fig. 5 shows the relationship between the percentage of lesions correctly identified (as found with each of the three reconstructions) and the distance from the correct lesion to the nearest alternative lesion site. The distance was measured between the centres of mass of all potential lesion sites for a given patient. As would be expected, there is an increase in the percentage of lesions correctly identified (i.e. lesions become more easily differentiable) as the distance between lesions increases. This was true regardless of the errors introduced and may be suggestive of which patients can most benefit from OP-MEG recordings. It is also noted that the differences in performance between reconstruction methods is largest when lesions are separated by less than 5 cm.

## Discussion

We explored how anatomical lesion information could be combined with OP-MEG to refine surgical decision making in epilepsy. We found that including the expected lesion location in the interpretation of the OP-MEG recording could have considerable benefits for accurately identifying active lesions, particularly for potential sites which were close together. We found that large errors in sensor geometry and gain could be robustly tolerated, motivating the use of flexible OPM arrays in future. However, when prior location information did not perfectly describe the locus of electrical activity, better lesion decisions were made by algorithms with looser (or no) prior information. The loosely constrained reconstruction, still informed by the anatomical prior but not restricted to it, was able to accommodate these source current differences and so provided a good balance between robustness to sensor and source assumption errors. This consistently outperformed an uninformed reconstruction and had the advantage of returning a confidence value (i.e. a probabilistic model comparison between lesion sites).

Practically, flexible OP-MEG helmets have a number of advantages, as they will fit a large number of people, saving time moving sensors between helmets, and could be lighter than a rigid alternative. They are often avoided due to concerns that errors in sensor positioning will impact source localisation accuracy but here we show that these effects are marginal and, when anatomical priors are available, can be minimised with appropriate source reconstruction.

While this paper has been framed around epilepsy surgery, the results could have implications beyond this sphere. In general, we have shown that including source priors can reduce the impact of sensor errors in source localisation, which is pertinent for any experiment with a strong hypothesis for a region of interest within the brain. This adds to an existing literature on the topic of anatomical priors in MEG more generally^55,56^.

There were, however, a number of assumptions made in these simulations. We assume that a flexible array incurs no additional noise penalty (e.g. from moving loose wires or sensors). Cross-talk in OP-MEG comes predominantly from a modulation signal (which is applied to all measuring axes of each OPM) and local, active magnetic shielding (applied to all sensor axes to minimise the background magnetic field at each OPM) leaking onto neighbouring sensors^29^. It is generally small, up to around 3% of the original signal for sensors separated by 2 cm^21^ (although values of 8% have been observed^57^) but variations in sensor spacing will lead to variations in cross-talk. Additionally, the SNR of the source signal was fixed at -20 dB. Based on recent empirical OP-MEG recordings^35,37,38^, this number seems reasonable and fixing the value avoided complications of increased SNR with source extent. However, it also meant that SNR was not dependent on source depth, as deeper sources were endowed with stronger signals. Fundamentally, we cannot quantify the true level of performance, which can only be assessed based on post-operative seizure freedom. Here we have sought to model realistic error sources in order to test different approaches in a controlled manner.

We observed that increasing the error on the sensor gain in particular had very little impact on outcome. This is most likely because we overly simplified the way in which these gain errors were applied. We assume that, in this flexible array, geometrical and gain errors are independent across sensors. This would not be the case in reality if, for example, there was an environmental magnetic field gradient across the sensor array. As sensor gain is dependent on background field, such a spatially dependent field gradient would mean that the sensor gain was position dependent and varied smoothly over the head^34^. It may also be that the range of gain errors was set too low. The maximum tested value of 5% approximately corresponds to a 2.5 nT field change for QuSpin OPMs, which would be large for a recording but certainly not unreasonable.

In these simulations, each error source was treated independently. However, there may be interactions between them which exacerbate the source reconstruction. As discussed, gain and sensor position or orientation errors are inevitably linked in reality, as the magnetic field determines the gain and is itself spatially dependent. Examining the impact of these interactions is left for future investigation.

We overcame the limitations of over-constraining our MSP priors by adding a region around the lesion COM. Volumetric imaging methods (where no cortical surface and hence no fixed source orientation is assumed) could be an alternative solution, as they may also be more robust to inaccuracies in the source priors. Removing the restriction on the source orientation would effectively give the model more degrees of freedom, and so, while a point on the edge of a lesion oriented differently to the COM may not be well modelled by a dipole at the lesion COM, a dipole at the COM allowed to be oriented in the same direction as the neighbouring edge dipole may be a far better model.

In this study, we have focussed on the use of OP-MEG to address the problem of inconclusive identification of the epileptogenic focus from other imaging modalities. Other imaging techniques could be used to address the same question and we have not sought to compare the many different options. Scalp EEG is of particular interest as it is also a non-invasive, direct measure of electrophysiology which is more readily available than OP-MEG. The main theoretical advantage of MEG over EEG is that the forward model is less affected by an individual’s anatomy. In this scenario, where we are comparing a small number of potential sources (< = 14), the constraints on the reconstruction may mean that EEG performs as well as OP-MEG. A future extension to the work could include using an alternative, more detailed forward model (such as a finite element model) to simulate both OP-MEG and EEG data for a range of different anatomies, to determine the degree of benefit OP-MEG could provide over EEG.

Here we have shown, as may be expected, that lesions which are distal to one another (> 4 cm apart) can be separated with OP-MEG with high accuracy (> 98% when sensor errors are included). Nearby lesions, less than 2 cm from one another were far less accurately separated (> 65% correctly identified for the worst sensor errors simulated here (20 degree rigid rotation)). This information could be used to suggest which patients may most benefit from an OP-MEG recording. It should also be considered within the context of epilepsy surgery, where resection volumes are often over 8 cm^3 ^^58^. Additionally, the simulated sources here were based on lesion sites identified from MRI by machine learning methods. Sites less than 2 cm from one another may be part of one larger lesion, where the centre has not been segmented. As such, separating very nearby lesions is perhaps less necessary. Nevertheless, as surgical techniques and precision improves, such decisions are likely to become more important. In such cases, it should be noted that it is at these distances (below 4 cm) where the information from anatomical priors has the most impact.

We tested three methods for differentiating simulated epileptiform lesions from inactive potential lesion sites: an uninformed method where the whole brain was the potential source space, a restricted model comparison method where the data was all assumed to come from the centre of one of the lesions, and a loosely constrained method where the current could come from any of the 100 cortical mesh vertices nearest to each lesion. This loosely constrained method provided a good compromise between adaptability to inaccurate source models and robustness to sensor errors. However, this region of 100 vertices was chosen arbitrarily. It could be increased in size to improve adaptability to inaccurate source models or decreased to improve robustness to sensor errors. Ideally, the size of the region would be optimised for the individual patient. It could instead be set based on the size of the lesion in MRI, or based on the accuracy and precision of the prior information. Alternatively, the size or importance of the priors could be altered based on the Free Energy of the source reconstruction^55^.

Here, the source reconstruction priors were selected based on suspected FCDs segmented from structural MRIs by the MELD algorithm. In practice, a similar approach could be taken, with potential sites segmented from MRI by either machine learning methods^59–61^ or by trained neuroradiologists. Information from other imaging modalities and clinical observation could also be used to decide which priors are chosen and their size, tailoring the OP-MEG source reconstruction to the individual.

It is worth noting that without any prior information, the correct lesion could still be separated from the other potential sites in the majority of cases, regardless of the errors introduced. In all cases, the percentage of lesions identified was well above chance level of 24.4%. Put another way, source reconstruction performance was improved by including prior information but was good even without it. This is expected based on previous OP-MEG recordings^22,25,27,37,62^, but is nevertheless encouraging for use of OP-MEG more widely in epilepsy planning.

At present, OP-MEG is an extremely new neuroimaging modality within the context of epilepsy. A handful of patients have been recorded from within a research setting, but, although much work is underway to further its clinical translation, there is currently minimal data available from which to determine the likely true success rate of the framework for identifying the epileptogenic focus set out within this manuscript. The simulation approach taken here allowed us to consider a large number of patients (> 500) with a range of different potential lesion sites.

These simulations suggest that OPMs could be a useful addition in the presurgical evaluation of epilepsy. We demonstrated that combining information from anatomical imaging with OP-MEG improved its performance, and that the impact of this information was greatest for distinguishing nearby lesions. Including such information also made the OP-MEG reconstruction more robust to errors in sensor position and orientation, which are present in realistic systems and may be exacerbated with flexible OPM helmets. We suggest that with considered use of prior information from other imaging modalities and clinical assessments, we can therefore overcome some of the limitations associated with generic OPM helmet designs. This has the potential to increase the clinical practicability of this promising new neuroimaging technology.

### Supplementary Information

Supplementary Information.

## Data Availability

Code required to reproduce the results in this manuscript, including to simulate the OP-MEG recordings, is available on GitHub (https://github.com/stephaniemellor/opm_meld_simulations). Requests for access to the MELD dataset can be made through the project website https://meldproject.github.io//.
