# Supplementary material for: Combining OPM and lesion mapping data for epilepsy surgery planning: a simulation study
Source: Sci Rep. 2024 Feb 4;14:2882. doi: 10.1038/s41598-024-51857-3 (PMC10838931; doi:10.1038/s41598-024-51857-3)
Supplement: Supplementary file 1 — Supplementary Information. [file 41598_2024_51857_MOESM1_ESM.docx]

Supplementary Material:

Combining OPM and lesion mapping data for epilepsy surgery planning: a simulation study

Stephanie Mellor^1^*, Ryan C. Timms^1^, George C. O’Neill^2^, Tim M. Tierney^1^, Meaghan E. Spedden^1^, The MELD Project Consortium, Matthew J. Brookes^3^, Konrad Wagstyl^1,4^, Gareth R. Barnes^1^

1. Wellcome Centre for Human Neuroimaging, UCL Queen Square Institute of Neurology, University College London, London WC1N 3AR, UK
2. Department of Neuroscience, Physiology and Pharmacology, University College London, London, UK
3. Sir Peter Mansfield Imaging Centre, School of Physics and Astronomy, University of Nottingham, Nottingham, UK
4. UCL Great Ormond Street Institute for Child Health, 30 Guilford St, University College London, London WC1N 1EH, UK

* Corresponding Author: Stephanie Mellor, [stephanie.mellor.17@ucl.ac.uk](mailto:stephanie.mellor.17@ucl.ac.uk)

# Dataset Simulation

OP-MEG data were simulated in SPM12. The sensor array, source mesh and simulated signal morphology and SNR are described in the main manuscript. Here we focus on how the different regions of a given lesion were simulated. In all cases, source activity was simulated using the Nolte single shell forward model (Nolte, 2003). Since SPM12 requires coordinates to be provided in MNI space, the lesion prediction coordinates in the space of the FreeSurfer template (fsaverage) were first transformed into MNI space using the transformation matrix found with SPM12.

## Centre of Mass (COM)

The cortical mesh vertex closest to the mean location of all predicted lesion coordinates (in MNI space) was simulated as active. This was simulated as a surface around the vertex but with a very small extent (FWHM 0.1 mm).

## Whole Lesion

The closest point on the cortical mesh to the MNI coordinates of each lesion prediction vertex was found. Repetitions of the same vertex (due to the downsampling of the cortical mesh for the simulation) were removed. Each vertex was then simulated as active, with an identical signal and strength. Each vertex was treated as a surface centred at the vertex but with a FWHM of 4.1196 mm to account for the spacing between mesh vertices.

## Whole Edge of the Lesion

As when defining the whole lesion, the closest cortical mesh vertices to each of the predicted lesion vertices were found. A boundary was then defined using the Matlab “convhull” function. To simulate activity from the boundary, the closest cortical mesh vertices to the boundary were found and were simulated as active, with identical signal and strength, with each vertex treated as a surface centred on the vertex with a FWHM of 4.1196 mm. It should be noted that due to the downsampling of the cortical mesh, this condition was the same as the whole lesion case for 64.5% of the simulated 1309 predicted lesions.

## Single dipole on the edge of the Lesion

The vertices of the cortical mesh describing the edge of the predicted lesion were found in the same way as described in section 1.3. From the set of edge vertices excluding, if present in the original set, the vertex closest to the centre of mass of the lesion (which was simulated as active in the COM condition in section 1.1), a random vertex was chosen using the Matlab “randi” function. A surface with FWHM of 4.1196 mm centred on the vertex was simulated as active. For one of the 1309 lesion predictions, due to the size of the lesion and the sampling of the cortical surface, only one vertex was found on the lesion edge and that vertex was also the COM of the lesion. For this one lesion prediction, the COM simulated data were copied to create the piece of edge data.

# Performance of uninformed (i.e. default) MSP


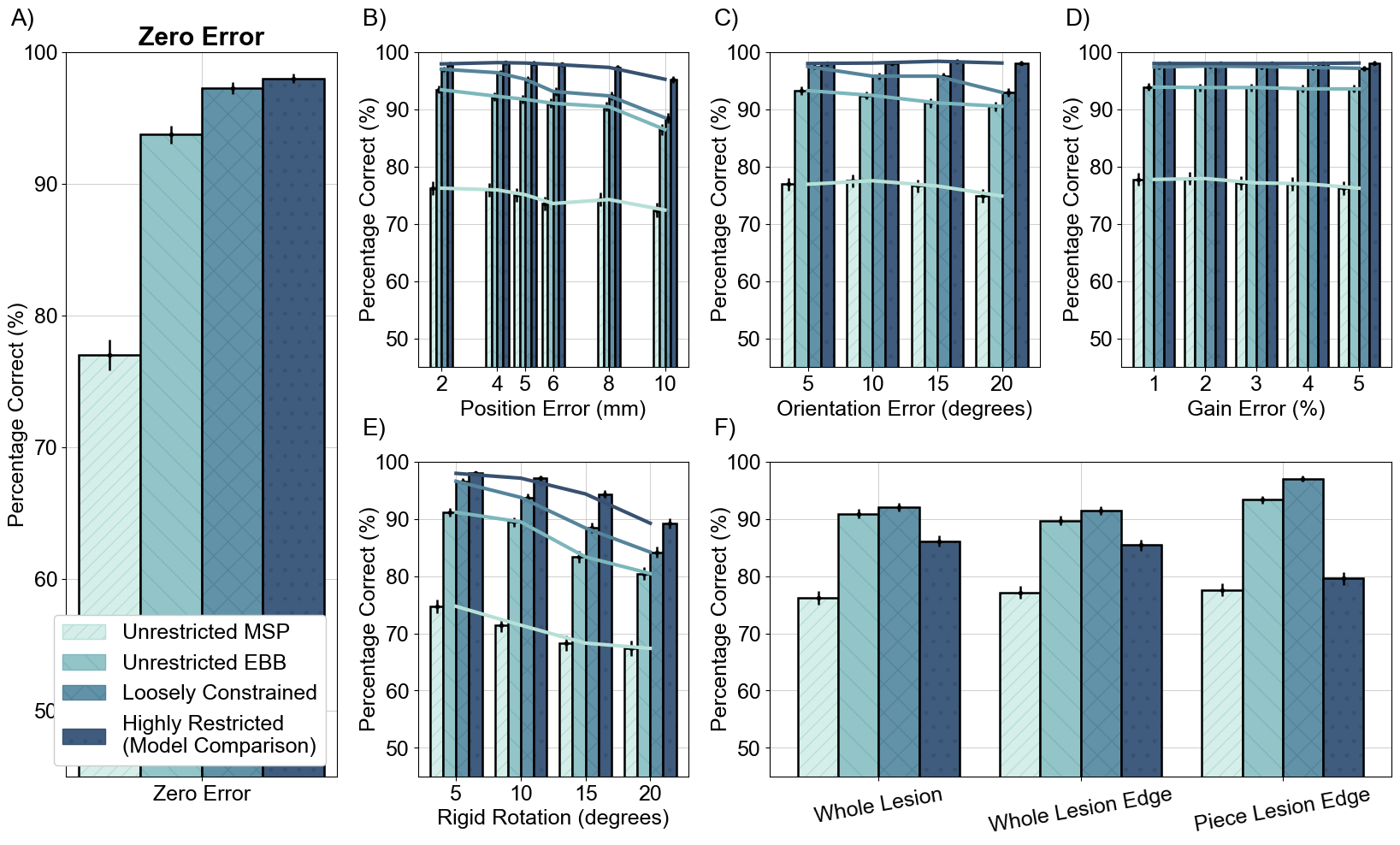


Supplementary Figure 1. Figure 4 of the main manuscript, with the addition of an unrestricted implementation of MSP, i.e. running MSP with the default settings in SPM. The reasons MSP performs less well than EBB in this case is that there are limited priors (512) which do not necessarily overlap with the simulated source location. Also, MSP relies on a non-linear (greedy) search to select the optimal prior set which can sometimes result in a local (rather than) global maximum in Free Energy (López, Espinosa and Barnes, 2012). One should note that this situation was ideal for the EBB algorithm (with a single source) but had we selected a more challenging case (with correlated sources) then its performance would also be degraded.

# Results with 25 simulated sensors


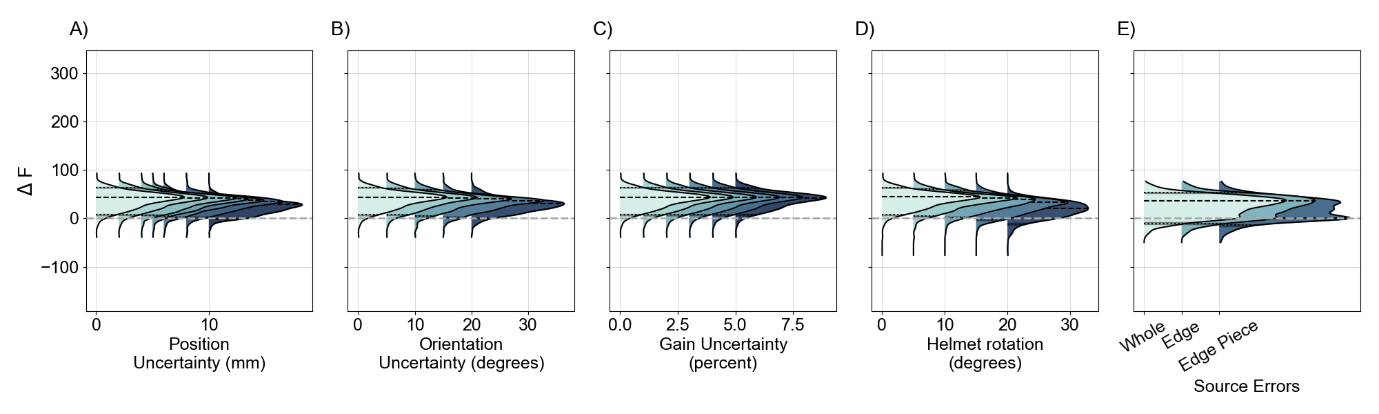


Supplementary Figure 2. Version of Figure 3 from the main manuscript with 25 OPM sensors. Distribution of the Free Energy difference between the correct (simulated) lesion model and the alternative lesion model with the next highest Free Energy ($\Delta F$) for all 1309 lesions from the restricted reconstruction. Values of $\Delta F$ below 0 indicate that an incorrect lesion would be selected. The maximum of the distribution and 5^th^ and 95^th^ percentile values are marked with dashed black lines. In (A)-(D), different sensor errors are shown. Flexible type errors (position (A), orientation (B) and gain uncertainties (C)) have a small effect by comparison with rigid helment rotation (D). Source errors (E), show the largest decrease in $\Delta F$ from the zero-error case.


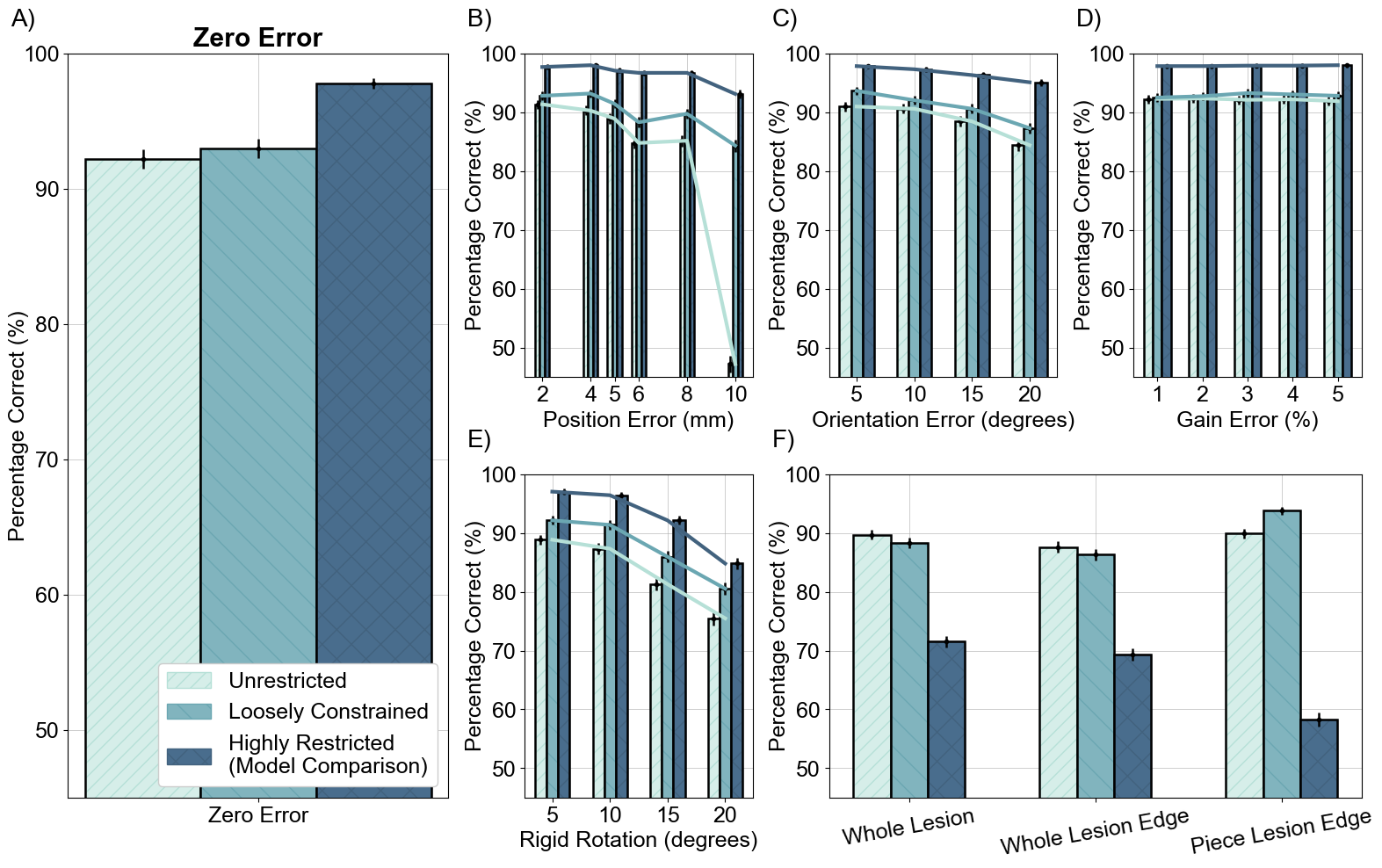


Supplementary Figure 3. Version of Figure 4 from the main manuscript with 25 OPM sensors. Comparison of the reconstruction methods tested: unrestricted EBB, MSP loosely constrained around the lesion site and MSP restricted to the centre of mass of the lesion. The percentage of lesions which were correctly differentiated from any of the patient’s other lesions is shown against the different errors added. By comparison to the case with 95 OPMs, the performance of all reconstructions is notably worse. The biggest difference is in panel F, where the unconstrained reconstruction outperforms the loosely constrained one when multiple dipoles were simultaneously active. This is likely due to the differences between EBB and MSP rather than including prior information. When only one dipole is active in the piece of lesion edge case, the loosely constrained method again outperforms the unrestricted one.


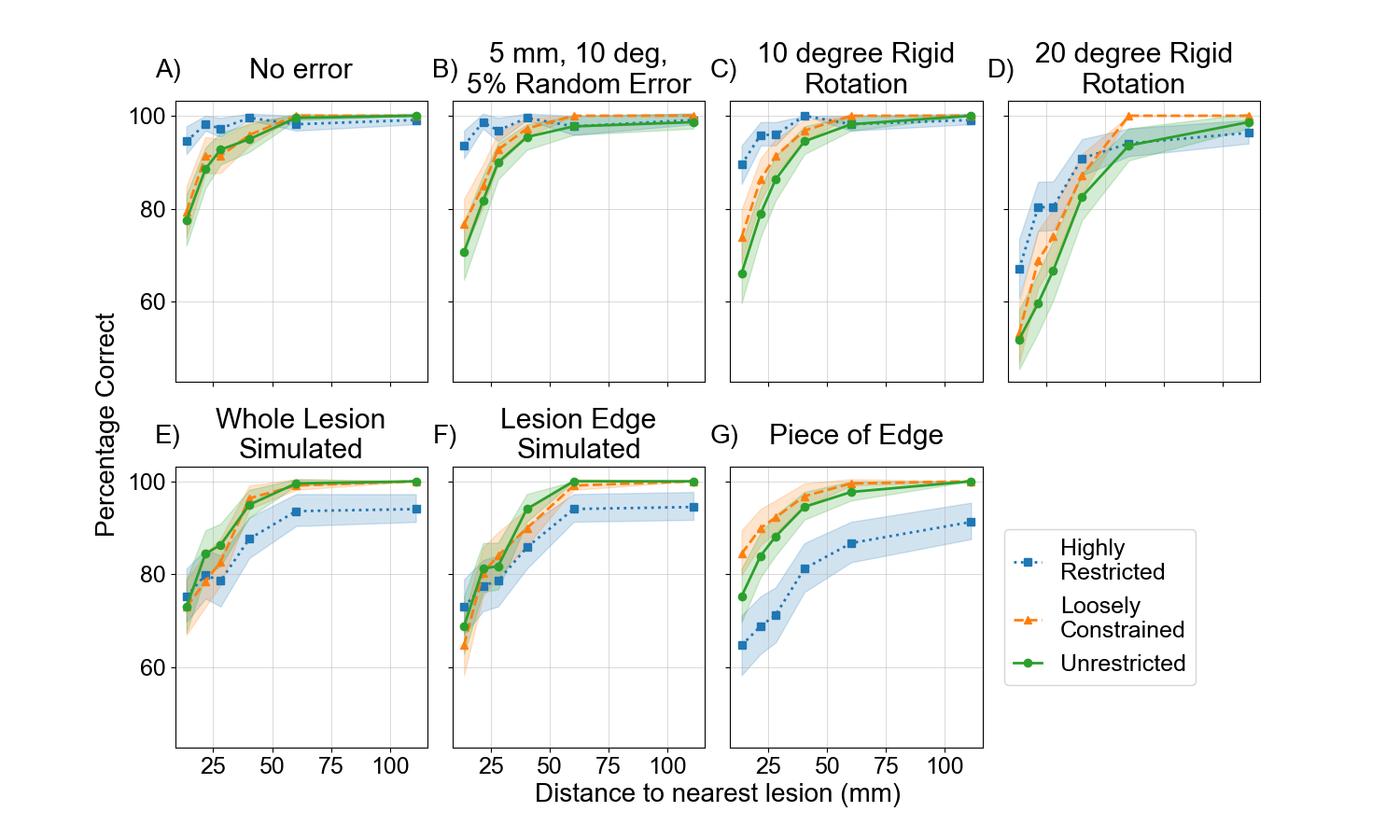


Supplementary Figure 4. Version of Figure 5 from the main manuscript with 25 OPM sensors. Relationship between the percentage of lesions correctly identified and distance to the nearest lesion from the same patient (i.e. the nearest alternative potential lesion site). All three reconstruction methods are shown for a selection of sensor and source errors. Each datapoint represents the percentage of lesions correctly identified within a distance range (or bin). The distance ranges are sampled so that there are equal numbers of lesions in each distance range (or bin). The points indicate the centre of each bin. The shaded area gives the 95% confidence interval of the percentage correct in each bin, estimated by bootstrapping with the scipy stats toolbox. For most of the panels, the relationships are the same as in the main manuscript but as in Supplementary Figure 3, the loosely constrained method does appear to struggle more when the whole lesion or whole of the lesion edge were simulated.

# Low SNR (only for patients with 2 lesions)

For all patients with only two lesions, we repeated the simulations with the SNR level fixed at -40 dB. At such a level, the signal was barely discernible from the background noise, even after the temporal projection in SPM. As only patients with 2 lesions were tested, this encompassed a subset of 190 lesions out of the total 1309.


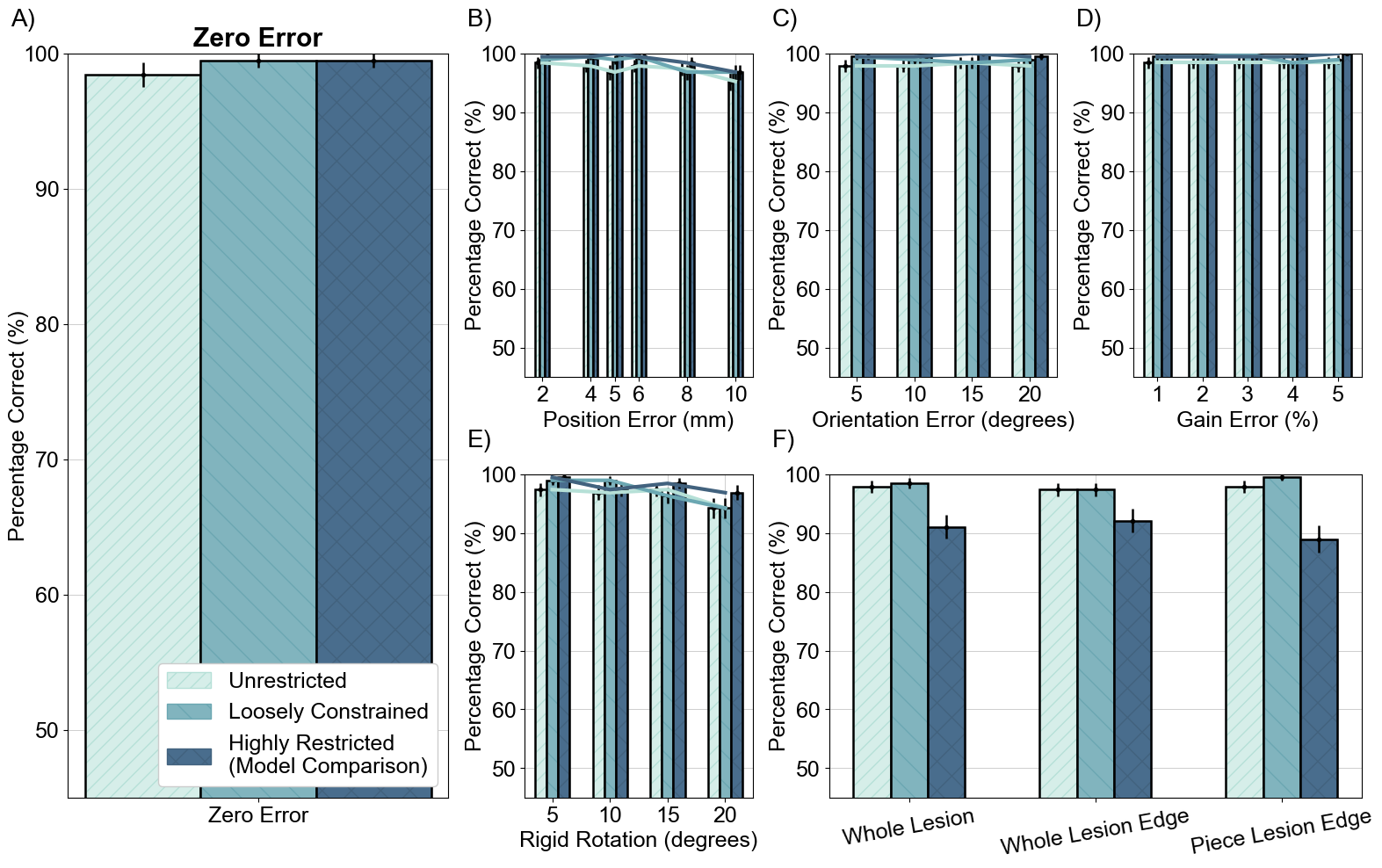


i)

ii)


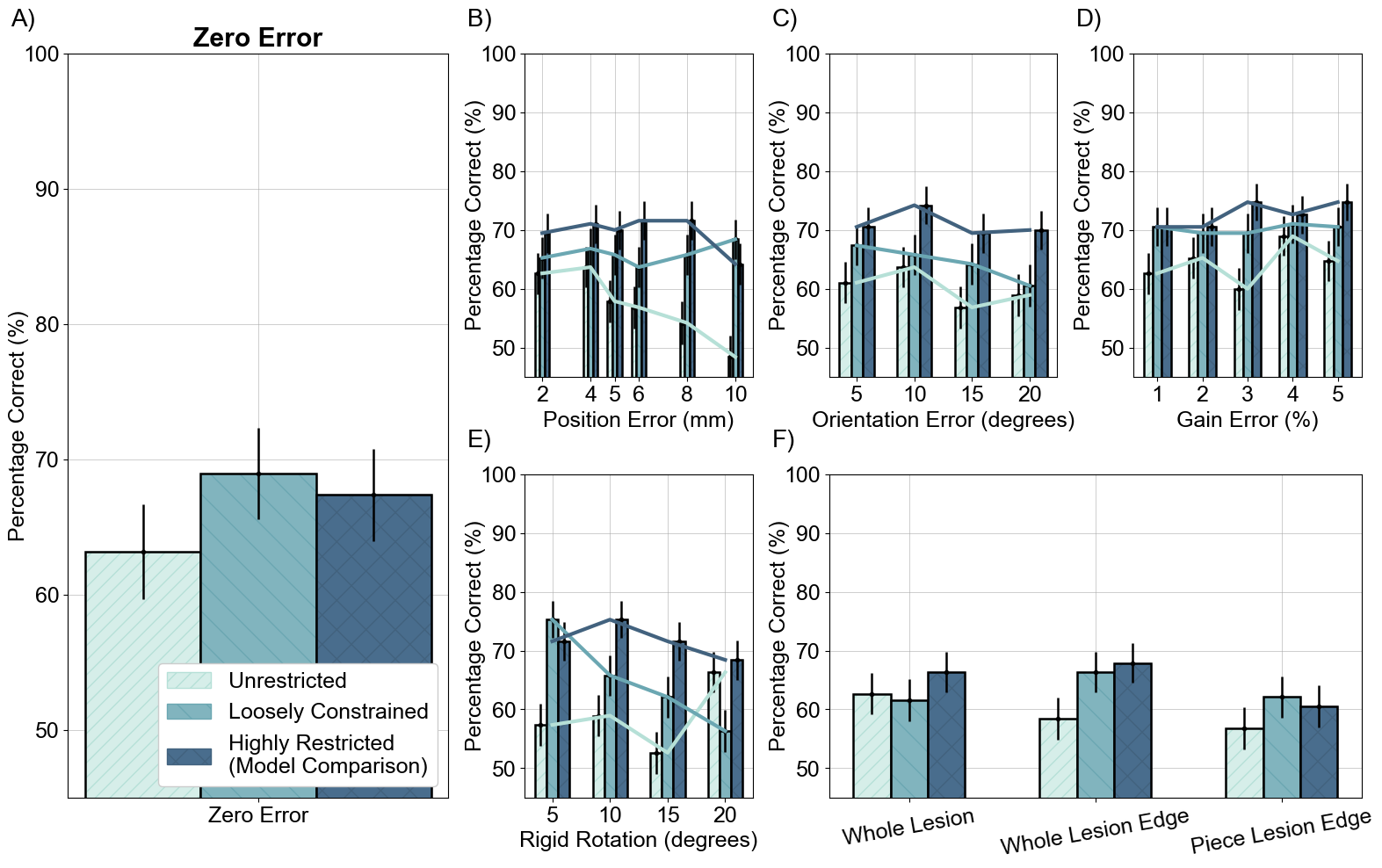


Supplementary Figure 5. Version of Figure 4 from the main manuscript with only patients with 2 lesions (i.e. chance level is 50%). In i) SNR is set at -20 dB, as for all the other simulations shown, while in ii), SNR is set at -40 dB. The percentage of lesions which were correctly differentiated from any of the patient’s other lesions is shown against the different errors added. In general, even at lower SNR, the loosely constrained method still provides a good compromise between sensitivity to source and sensor errors. However, the low SNR does make the methods considerably harder to distinguish.

# References

López, J.D., Espinosa, J.J. and Barnes, G.R. (2012) ‘Random location of multiple sparse priors for solving the MEG/EEG inverse problem’, in *2012 Annual International Conference of the IEEE Engineering in Medicine and Biology Society*. *2012 Annual International Conference of the IEEE Engineering in Medicine and Biology Society*, pp. 1534–1537. Available at: https://doi.org/10.1109/EMBC.2012.6346234.

Nolte, G. (2003) ‘The magnetic lead field theorem in the quasi-static approximation and its use for magnetoencephalography forward calculation in realistic volume conductors’, *Physics in Medicine & Biology*, 48(22), p. 3637. Available at: https://doi.org/10.1088/0031-9155/48/22/002.
